# The role of osteoclasts in breast cancer bone metastasis

**DOI:** 10.1016/j.jbo.2016.02.008

**Published:** 2016-04-08

**Authors:** François Le Pape, Geoffrey Vargas, Philippe Clézardin

**Affiliations:** a INSERM, UMR 1033, Lyon F-69372, France; b Université Lyon-1, Villeurbanne F-69622, France

**Keywords:** RANKL, RANK, OPG, PTHrP, miRNA, Bone resorption

## Abstract

Breast cancer frequently metastasises to the skeleton, interfering with the normal bone remodelling process and inducing bone degradation. Bone degradation is caused by osteoclasts, the normal bone-resorbing cells. Osteoclast-mediated bone degradation subsequently leads to the release of bone-derived factors that promote skeletal tumour growth. Osteoclasts themselves stimulate tumour growth. This Review describes the molecular mechanisms through which osteoclasts and breast cancer cells collaborate with each other, triggering the formation of osteolytic bone metastasis.

## Highlights

• RANK-RANKL-OPG pathway regulates osteoclast formation, function and survival.
• Cancer-mediated osteolysis is caused by osteoclast stimulation and osteoblast inhibition.
• Bone matrix-embedded growth factors are released upon bone degradation.
• Growth factors released from resorbed bone stimulate tumour growth.

## 1 Introduction

Once metastatic breast cancer cells are in the bone marrow, they hijack signals coming from the normal bone remodelling process and promote bone degradation [1]. Bone degradation is caused by osteoclasts, which have the unique property to dissolve bone mineral and degrade the bone matrix. These features make them a predominant actor in bone metastasis formation. This review describes how osteoclasts contribute to bone degradation and skeletal tumour growth.

## 2 Bone physiology

### 2.1 Osteoclastogenesis

Osteoclasts derive from haematopoietic stem cells. Osteoclast differentiation is dependent on two key molecules, Macrophage Colony Stimulating Factor (M-CSF) and Receptor activator of NFκB ligand (RANKL), the latter being also a mediator of osteoclast function and survival [2], [3]. The stimulation of the RANK pathway by RANKL is negatively regulated by a decoy receptor, osteoprotegerin (OPG), which is expressed by osteoblasts [3]. Others factors that influence osteoclast formation are Tumour Necrosis Factors α (TNFα) [4] and interleukins (IL-1, IL-8, IL-11) [5].

### 2.2 Osteoclast-mediated bone resorption

Osteoclasts are the normal bone-resorbing cells. Osteoclast-mediated bone resorption can be viewed as a sequential process where dissolution of the mineral phase of bone precedes matrix degradation. Bone demineralization involves the secretion of protons by osteoclasts which, in turn, provide an optimal acidic microenvironment for the proteolytic activity of osteoclast-derived cathepsin K, enabling degradation of the demineralized collagenous matrix [5]. Matrix metalloprotease MMP13 is also involved in the degradation of the collagen matrix [6]. This degradation leads to the release of calcium (Ca^2^^+^) and of bone-derived growth factors embedded within the bone matrix, such as Transforming Growth Factor (TGFβ), Insulin Growth Factors (IGFs), and Platelet derived growth factor (PDGF) [7].

## 3 Osteolytic bone metastasis: the “Vicious Cycle”

**Fig. 1:**
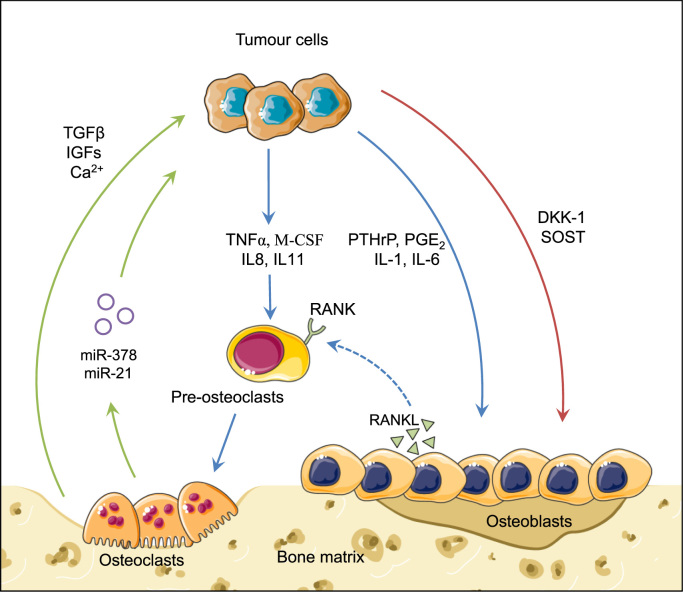
Breast cancer bone metastasis formation. In bone**,** breast tumour cells secrete different factors that enhance osteoclast differentiation and activity (blue arrows). Consequently, bone-resorbing activity of mature osteoclasts is increasing. Bone-embedded growth factors, which are released from the bone matrix, and miRNAs secreted by osteoclasts then promote tumour growth (green arrows). In addition, tumour cells secrete factors (DKK-1, SOST) that inhibit osteoblast differentiation and activity, thereby contributing to cancer-induced bone destruction (red arrows).

Once metastatic cancer cells colonize bone, they do not, on their own, destroy bone. They interact with osteoblasts, the normal bone-forming cells, and osteoclasts to induce massive bone degradation. In turn, bone-derived growth factors and calcium released from resorbed bone stimulate skeletal tumour growth. This relationship between bone resorption and tumour growth is called the “vicious cycle” (Fig. 1). This is the reason why anti-resorptive drugs such as bisphosphonates and denosumab, a human monoclonal antibody directed against RANKL, are used in clinic, as palliative treatment, to interfere with this vicious cycle [8].

### 3.1 Tumour-derived factors that promote osteoclast-mediated bone degradation

Several molecules that are produced by breast cancer cells, such as interleukins (IL-8, IL-11), M-CSF, and TNFα directly stimulate osteoclast activity [9]. Other factors including Parathyroid Hormone-related Protein (PTHrP), interleukins (IL-1, and IL-6) and prostaglandin E_2_ (PGE_2_) enhance osteoclast formation through the regulation of RANKL/OPG production by osteoblasts [10]. In sum, tumour cells produce a large panel of soluble factors that promote bone degradation (Fig. 1). However, tumour cells may produce factors that inhibit osteoclast activity [endothelin-1 (ET-1), OPG], leading to the formation of osteoblastic or mixed lesions [11], [12].

### 3.2 Tumour-derived factors that contribute to bone degradation

Cathepsin K is produced by tumour cells [13]. It promotes tumour cell invasiveness and may also contribute to bone degradation [13]. In this respect, there is preclinical evidence that treatment of animals bearing bone metastases with a cathepsin K inhibitor (odanacatib, AFG-495) partially blocks bone degradation [13]. Additionally, a number of factors that inhibit osteoblast differentiation [dickkopf-1 (DKK-1) and sclerostin (SOST)] have been reported to be secreted by tumour cells [9]. By inhibiting bone formation, DKK-1 and SOST will also indirectly contribute to bone degradation (Fig. 1).

### 3.3 Osteoclast-derived factors that promote tumour growth

MicroRNAs (miRNAs) expressed by tumour cells can act as master regulators of bone metastasis formation [14]. Although osteoclasts are mostly known for being directly involved in mediating bone resorption, they also secrete miRNAs that modulate skeletal tumour growth [15], [16] (Fig. 1). For example, osteoclasts secrete exosomes containing miRNAs such as miR-378 which promotes tumour growth, angiogenesis and tumour cell survival through the repression of tumour suppressors SuFu and Fus-1 [17]. Additionally, osteoclast-derived miR-21 enhances tumour cell proliferation [18].

### 3.4 Factors released from resorbed bone that promote tumour growth

Bone is a reservoir of growth factors and calcium capable of stimulating growth of tumour cells (Fig. 1). Activated TGFβ stimulates tumour growth and PTHrP expression by tumour cells, which in turn stimulates osteoclast-mediated bone resorption [19]. IGFs, PDGF and BMP (Bone Morphogenic Protein) family members are also released from the bone matrix and they enhance tumour cell proliferation [9]. Calcium acts on tumour cells expressing the calcium-sensing receptor (CASR) by enhancing tumour cell survival [20].

## 4 Conclusion

Current knowledge on the pathogenesis of bone metastasis demonstrates that osteoclasts are playing a key role in mediating bone degradation and promoting skeletal tumour growth. These findings provide the rationale for using anti-resorptive drugs (bisphosphonates, anti-RANKL) in the treatment of bone metastasis. However, these molecules do not treat cancer but slow down its progression by limiting the extent of bone destruction. Thus, it's truly vital to increase our understanding of the cellular and molecular mechanisms that precede the overt development of skeletal lesions in order to develop novel therapeutic strategies.
